# Supplementary material for: The evolution of human population distance to water in the USA from 1790 to 2010
Source: Nat Commun. 2019 Jan 25;10:430. doi: 10.1038/s41467-019-08366-z (PMC6347611; doi:10.1038/s41467-019-08366-z)
Supplement: Supplementary file 1 — Supplementary Information [file 41467_2019_8366_MOESM1_ESM.docx]

Supplementary Information for

The Evolution of Human Population Distance to Water in the USA from 1790 to 2010

Fang and Jawitz

**This file includes:**

Supplementary Figures

Supplementary Tables

Supplementary Note

Supplementary References

Supplementary Figures

Supplementary Fig. 1. Human distance (D_H_) and geographical distance (D_G_) for HUCs. a The ratio of D_H_ and D_G_ to major rivers as a function of mean annual precipitation for the 19 HUCs in the conterminous US. b Accumulative percentage of land area and human population as a function of distance to major rivers for HUCs 3 and 14. The data points for b are based on DMR classes.


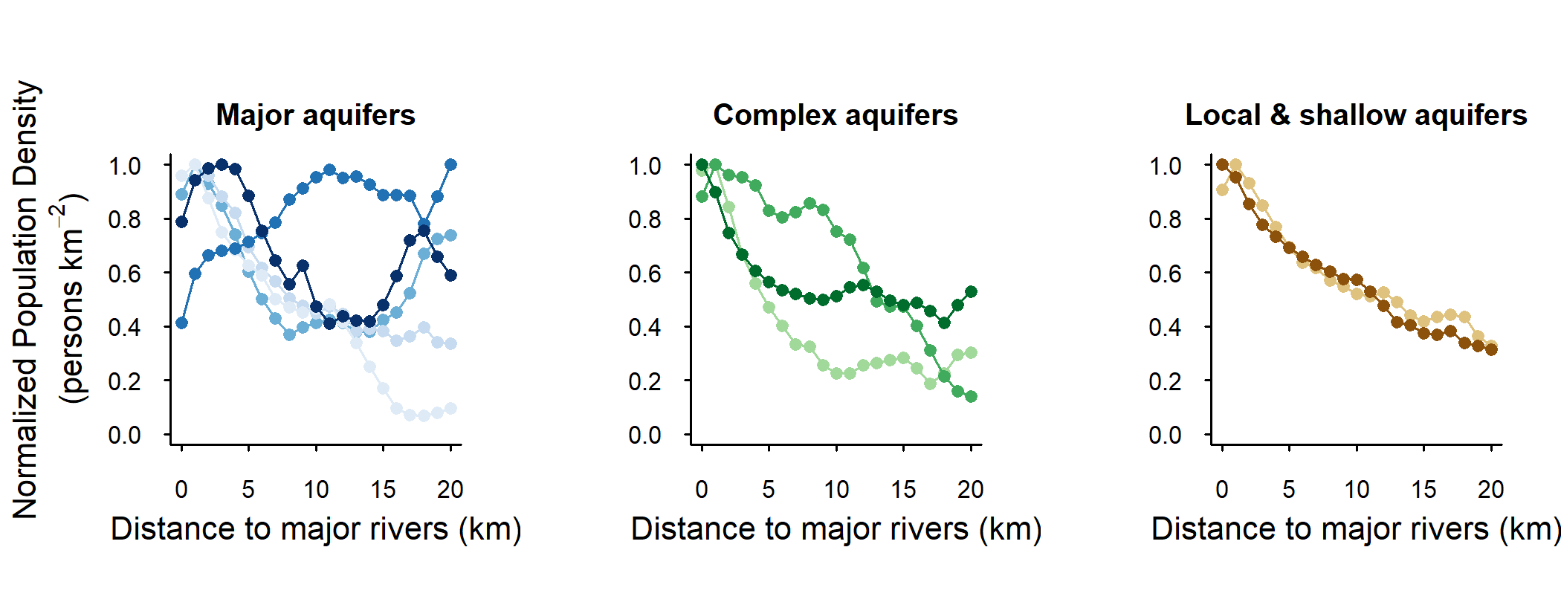
Supplementary Fig. 2. Population density with distance to rivers by aquifer type. Normalized population density is shown as a function of distance to major rivers for different types of aquifers in the entire conterminous US in 2010. For each type of aquifer, darker colors indicate higher groundwater recharge rates.

(Continued)

Supplementary Fig. 3. Desirability of living close to major rivers for HUCs. Different colors show varied trajectories: black (HUC 1, 2, 11, and 12) indicates stable desirability, red (HUC 3, 7, 8, 1718) indicates decreasing desirability, blue (HUC 4, 6, 9, 13, 14, 15, 16, 17A) indicates increasing desirability, while orange (HUC 5) and light blue (HUC 10, 18A) mean combination of trends. Desirability of living close to major rivers is measured by the slope of population density versus distance.


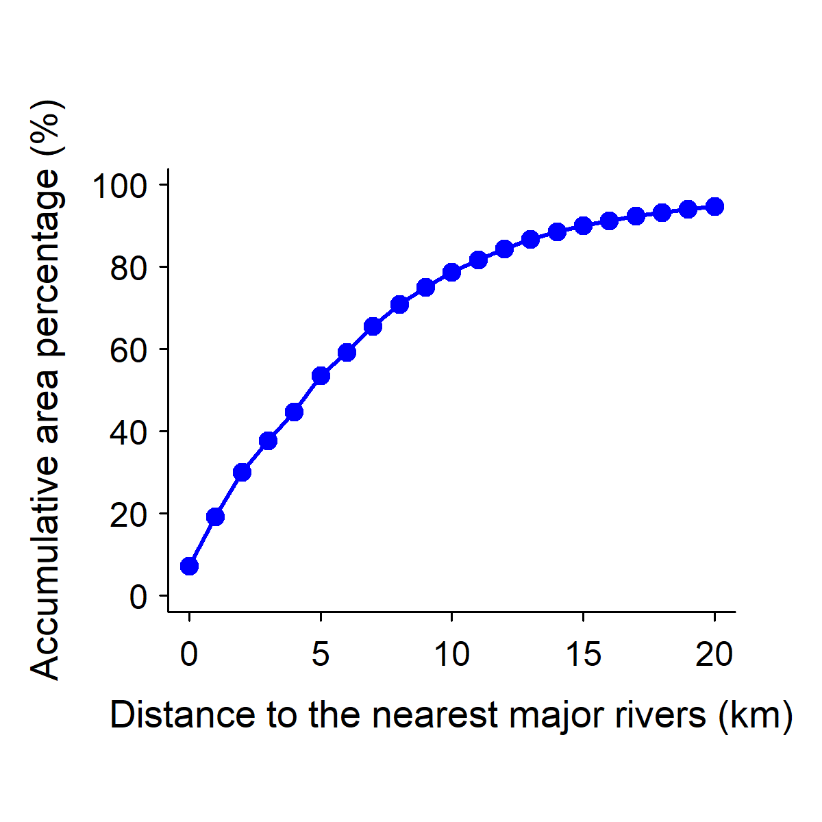


Supplementary Fig. 4. Accumulative area percentage for the entire conterminous US. Catchment contributing area increases with distance from major rivers.

Supplementary Fig. 5. Spatio-temporal patterns of reservoir construction. a The trend of different types of reservoirs in the entire conterminous US over time. b Histogram of reservoir area percentage within river courses in the entire conterminous US.

Supplementary Fig. 6. Coefficient of variation of climate variables for HUCs. Mean annual precipitation and mean temperature are shown for 18 HUC regions in the entire conterminous US.


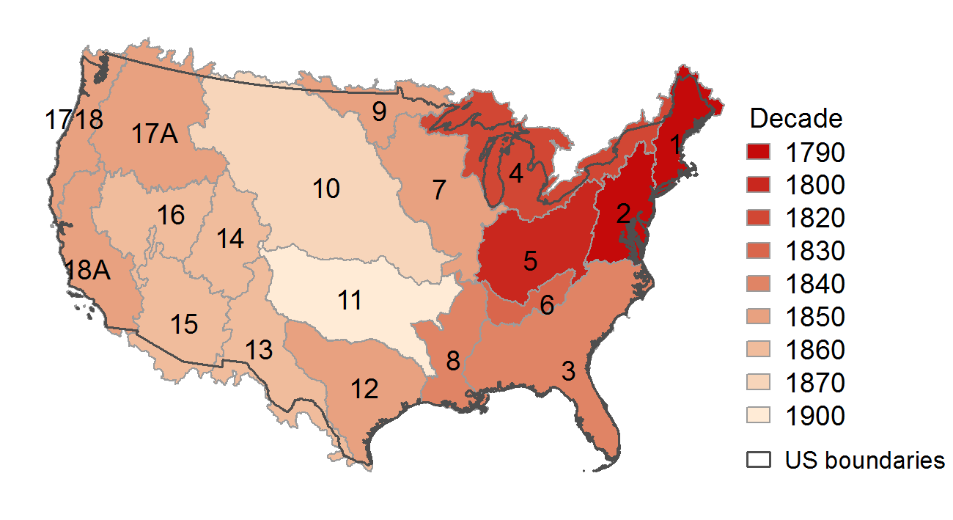


Supplementary Fig. 7. The initial decade of analysis for each HUC.

Supplementary Tables

Supplementary Table 1. Increasing trend of mean population density from $\boldsymbol{t}_{\mathbf{0}}$ to 2010 for each aquifer type.

| Aquifer types | | Area percentage (%) | $r_{0}$ | $r$ | $t_{0}$ | R^2^ | *p* value |
| --- | --- | --- | --- | --- | --- | --- | --- |
| Groundwater zone | Recharge rate (mm per year) |  |  |  |  |  |  |
| **Major groundwater basins** | | | | |  |  |  |
| 11 | <2 | 5.8 | 0.101 | 1.036 | 1860 | 0.99 | 0.00 |
| 12 | 2-20 | 8.4 | 0.126 | 1.032 | 1850 | 0.94 | 0.00 |
| 13 | 20-100 | 4.1 | 0.642 | 1.027 | 1850 | 0.90 | 0.00 |
| 14 | 100-300 | 10.9 | 2.661 | 1.017 | 1800 | 0.99 | 0.00 |
| 15 | ≥300 | 0.4 | 0.698 | 1.043 | 1850 | 0.88 | 0.00 |
| **Aquifers with complex hydrogeological structures** | | | | |  |  |  |
| 22 | <20 | 14.1 | 0.237 | 1.025 | 1870 | 0.90 | 0.00 |
| 23 | 20-100 | 4.9 | 0.575 | 1.029 | 1820 | 0.81 | 0.00 |
| 24 | 100-300 | 14.6 | 2.337 | 1.018 | 1800 | 0.91 | 0.00 |
| **Local and shallow aquifers** | | | | |  |  |  |
| 33 | <100 | 20.3 | 0.472 | 1.030 | 1850 | 0.87 | 0.00 |
| 34 | ≥100 | 16.6 | 3.930 | 1.013 | 1800 | 0.98 | 0.00 |

Supplementary Table 2. Slope of NPD vs DMR in 2010 for each aquifer type

| Aquifer types | | Area percentage (%) | slope | R^2^ | *p* value |
| --- | --- | --- | --- | --- | --- |
| Groundwater zone | Recharge rate (mm per year) |  |  |  |  |
| **Major groundwater basins** | | | | | |
| 11 | <2 | 5.8 | -0.047 | 0.95 | 0.00 |
| 12 | 2-20 | 8.4 | -0.034 | 0.86 | 0.00 |
| 13 | 20-100 | 4.1 | -0.015 | 0.20 | 0.04 |
| 14 | 100-300 | 10.9 | 0.019 | 0.60 | 0.00 |
| 15 | ≥300 | 0.4 | -0.020 | 0.37 | 0.00 |
| **Aquifers with complex hydrogeological structures** | | | | | |
| 22 | <20 | 14.1 | -0.033 | 0.64 | 0.00 |
| 23 | 20-100 | 4.9 | -0.043 | 0.91 | 0.00 |
| 24 | 100-300 | 14.6 | -0.018 | 0.58 | 0.00 |
| **Local and shallow aquifers** | | | | | |
| 33 | <100 | 20.3 | -0.030 | 0.90 | 0.00 |
| 34 | ≥100 | 16.6 | -0.033 | 0.95 | 0.00 |

Supplementary Table 3. Mean population density in 2010 by climatic zone for aquifers.

| Aquifer types | | Mean population density (persons km^-2^) | | | | | |
| --- | --- | --- | --- | --- | --- | --- | --- |
| groundwater zone | Recharge rate (mm per year) | whole conterminous US | Tropical zone | Warm Temperate zone | Cool Temperate zone | Polar zone | Boreal zone |
| **Major groundwater basins** | | | | | | | |
| 11 | <2 | 17.2 | 67.5 | 11.2 | 3.4 | - | - |
| 12 | 2-20 | 13.1 | 28.2 | 11.7 | 3.0 | - | - |
| 13 | 20-100 | 34.5 | 58.0 | 41.7 | 16.5 | - | - |
| 14 | 100-300 | 73 | 58.5 | 77.2 | 36.2 | - | - |
| 15 | ≥300 | 230.9 | - | 324.4 | 36.2 | - | - |
| **Aquifers with complex hydrogeological structures** | | | | | | | |
| 22 | <20 | 5.4 | 4.4 | 6.9 | 6.0 | 0.08 | 0.35 |
| 23 | 20-100 | 51.9 | 133.5 | 58.4 | 37.4 | 0.23 | 1.88 |
| 24 | 100-300 | 58.2 | 120.5 | 59.5 | 51.2 | 4.66 | - |
| **Local and shallow aquifers** | | | | | | | |
| 33 | <100 | 29.6 | 166.6 | 43.7 | 7.1 | 0.63 | 0.72 |
| 34 | ≥100 | 53.2 | 18.7 | 63.0 | 42.7 | - | 1.56 |

Supplementary Table 4. Lateral migration rate of rivers collected from previous studies.

| **No.** | **River** | **Lateral migration rate** | **Source** |
| --- | --- | --- | --- |
| 1 | Powder River | Maximum: 5 m per year (1830-2014) | 44 |
| 2 | Rio Grande, New Mexico | 0.25-1.44 m per year (1992-2001) | 45 |
| 3 | Brazos River, Texas | 1.18-3.39 m per year (1989-2011) | 46 |
| 4 | the Nueces River in Texas | 0.95-1.51 m per year (1962-2012) |  |
| 5 | the Sabine River in Texas | 0.77-2.98 m per year (1989-2011) |  |
| 6 | the Trinity River in Texas | 0.69-2.5 m per year (1957-2009) |  |
| 7 | Milk River | Decreased from 1.7 m per year to 0.46 m per year after dam closure | 47 |
| 8 | lower Mississippi River | 45.2 m per year and 59.1 m per year in the upper and lower alluvial valley (1877-1924) | 48 |
| 9 | Congaree River, South Carolina | 0.1-1.8 m per year, with 0.29 m per year for the upper and 1.14 m per year for the lower reach (1938-2006) | 49 |
| 10 | Winooski River, Vermont | 1.0 m per year | 50 |
| 11 | Connecticut River, Vermont | 4.1 m per year |  |
| 12 | Genesee River, New York | 5.0 m per year |  |
| 13 | Missouri River, downstream from Fort Peck Dam, Montana | Decreased from 6.6 m per year to 1.8 m per year after impoundment (1890-1991) | 51 |
| 14 | Yellow River, China | 5.0, 7.9, -5.9, 8.6, and -1.0 m per year, during the periods 1975–1990, 1990–2000, 2000–2006, 2006–2010, and 2010–2011 | 52 |
| 15 | River Diana, sub-Himalayan West Bengal | 27.25 m per year (1929-2014) | 53 |
| 16 | One reach of the Ebro River, Spain, between Rincón de Soto (La Rioja) and the small dam of Alforque (La Zaida, Zaragoza) | 3.74 m per year (1927-1956) and 0.87 m per year (1957-2003) | 54 |
| 17 | Klip River, South Africa | 0.16 m per year (~160 m over ~1000 years) | 55 |
| 18 | Upper Amazon river | Maximum: 125 m per year (1986-2006) | 56 |

Supplementary Table 5. Relationship between lateral migration rate (M, m per year) and river width (W, m) from previous studies.

| **No.** | **M vs. W** | **M (W = 1000 m)** | **Source** |
| --- | --- | --- | --- |
| 1 | M = -0.275 + 0.00691W (r= -0.358, post-dam) | 6.64 m per year | 51 |
| 2 | M = 0.01W | 10.0 m per year | 57 |
| 3 | M = 0.28W^0.34^ (R^2^ = 0.34) | 2.93 m per year | 58 |
| 4 | M = 0.004W^1.189^ (R^2^ = 0.47) | 14.8 m per year | 45 |

Supplementary Table 6. Settlement area percentage (%) for each HUC in the conterminous US, from 1790 to 2010.

| Decade | HUC1 | HUC2 | HUC3 | HUC4 | HUC5 | HUC6 | HUC7 | HUC8 | HUC9 | HUC10 | HUC11 | HUC12 | HUC13 | HUC14 | HUC15 | HUC16 | HUC17 | HUC18 |
| --- | --- | --- | --- | --- | --- | --- | --- | --- | --- | --- | --- | --- | --- | --- | --- | --- | --- | --- |
| 1790 | 99.9 | 99.9 | 38.5 | 20.8 | 48.9 | 13.1 | 0.0 | 1.4 | 0.0 | 0.0 | 0.0 | 0.0 | 0.0 | 0.0 | 0.0 | 0.0 | 0.0 | 0.0 |
| 1800 | 100.0 | 99.9 | 39.1 | 100.0 | 97.7 | 34.9 | 49.7 | 5.4 | 13.5 | 0.0 | 0.0 | 0.0 | 0.0 | 0.0 | 0.0 | 0.0 | 0.0 | 0.0 |
| 1810 | 100.0 | 100.0 | 44.3 | 78.7 | 90.0 | 59.2 | 53.7 | 47.4 | 13.5 | 4.0 | 14.1 | 0.1 | 0.0 | 0.0 | 0.0 | 0.0 | 0.0 | 0.0 |
| 1820 | 100.0 | 99.9 | 63.6 | 90.3 | 97.3 | 82.7 | 51.8 | 71.8 | 13.5 | 6.7 | 17.6 | 1.3 | 0.0 | 0.0 | 0.0 | 0.0 | 0.0 | 0.0 |
| 1830 | 100.0 | 100.0 | 87.2 | 100.0 | 99.2 | 89.0 | 57.0 | 85.3 | 13.5 | 7.2 | 20.9 | 1.3 | 0.0 | 0.0 | 0.0 | 0.0 | 0.0 | 0.0 |
| 1840 | 100.0 | 100.0 | 100.0 | 92.3 | 100.0 | 100.0 | 81.9 | 99.9 | 33.4 | 8.0 | 19.3 | 1.3 | 0.0 | 0.0 | 0.0 | 0.0 | 0.0 | 0.0 |
| 1850 | 100.0 | 100.0 | 100.0 | 100.0 | 100.0 | 100.0 | 92.0 | 100.0 | 98.5 | 20.2 | 38.2 | 96.7 | 58.4 | 7.3 | 2.0 | 11.6 | 97.5 | 96.8 |
| 1860 | 100.0 | 99.9 | 100.0 | 96.6 | 100.0 | 100.0 | 98.8 | 99.9 | 46.6 | 28.2 | 44.4 | 73.6 | 94.3 | 96.3 | 100.0 | 96.2 | 99.8 | 100.0 |
| 1870 | 100.0 | 100.0 | 100.0 | 97.1 | 100.0 | 100.0 | 100.0 | 99.9 | 89.7 | 96.8 | 49.6 | 84.6 | 100.0 | 98.8 | 94.0 | 98.8 | 100.0 | 100.0 |
| 1880 | 100.0 | 99.9 | 100.0 | 100.0 | 100.0 | 100.0 | 100.0 | 99.9 | 80.6 | 93.2 | 69.0 | 94.8 | 99.8 | 100.0 | 100.0 | 100.0 | 100.0 | 100.0 |
| 1890 | 100.0 | 99.9 | 100.0 | 100.0 | 100.0 | 100.0 | 100.0 | 99.9 | 99.2 | 98.1 | 75.6 | 98.5 | 100.0 | 100.0 | 100.0 | 100.0 | 99.7 | 100.0 |
| 1900 | 100.0 | 99.9 | 100.0 | 100.0 | 100.0 | 100.0 | 100.0 | 99.9 | 100.0 | 100.0 | 99.7 | 100.0 | 100.0 | 100.0 | 100.0 | 100.0 | 100.0 | 100.0 |
| 1910 | 100.0 | 99.9 | 100.0 | 100.0 | 100.0 | 100.0 | 100.0 | 99.9 | 100.0 | 100.0 | 100.0 | 100.0 | 100.0 | 100.0 | 100.0 | 100.0 | 100.0 | 100.0 |
| 1920 | 100.0 | 99.9 | 100.0 | 100.0 | 100.0 | 100.0 | 100.0 | 99.9 | 100.0 | 100.0 | 100.0 | 100.0 | 100.0 | 100.0 | 100.0 | 100.0 | 100.0 | 100.0 |
| 1930 | 100.0 | 99.9 | 100.0 | 100.0 | 100.0 | 100.0 | 100.0 | 99.9 | 100.0 | 100.0 | 100.0 | 100.0 | 100.0 | 100.0 | 100.0 | 100.0 | 100.0 | 100.0 |
| 1940 | 100.0 | 99.9 | 100.0 | 100.0 | 100.0 | 100.0 | 100.0 | 99.9 | 100.0 | 100.0 | 100.0 | 100.0 | 100.0 | 100.0 | 100.0 | 100.0 | 100.0 | 100.0 |
| 1950 | 100.0 | 99.9 | 100.0 | 100.0 | 100.0 | 100.0 | 100.0 | 99.9 | 100.0 | 100.0 | 100.0 | 100.0 | 100.0 | 100.0 | 100.0 | 100.0 | 100.0 | 100.0 |
| 1970 | 100.0 | 99.9 | 100.0 | 100.0 | 100.0 | 100.0 | 100.0 | 100.0 | 100.0 | 100.0 | 100.0 | 100.0 | 100.0 | 100.0 | 100.0 | 99.9 | 100.0 | 100.0 |
| 1980 | 100.0 | 99.9 | 100.0 | 100.0 | 100.0 | 100.0 | 100.0 | 100.0 | 100.0 | 99.9 | 100.0 | 100.0 | 99.9 | 100.0 | 100.0 | 99.9 | 99.9 | 100.0 |
| 1990 | 100.0 | 99.8 | 100.0 | 100.0 | 100.0 | 100.0 | 100.0 | 100.0 | 100.0 | 100.0 | 100.0 | 100.0 | 100.0 | 100.0 | 100.0 | 100.0 | 100.0 | 100.0 |
| 2000 | 100.0 | 99.9 | 100.0 | 100.0 | 100.0 | 100.0 | 100.0 | 100.0 | 100.0 | 100.0 | 100.0 | 100.0 | 100.0 | 100.0 | 100.0 | 100.0 | 100.0 | 100.0 |
| 2010 | 100.0 | 99.9 | 100.0 | 100.0 | 100.0 | 100.0 | 100.0 | 100.0 | 100.0 | 100.0 | 100.0 | 100.0 | 100.0 | 100.0 | 100.0 | 100.0 | 100.0 | 100.0 |

Note. The starting year of analysis was determined for each HUC as the year when the area percentage reached 90%.

Supplementary Note

River channel migration is the movement of a river channel back and forth across its valley in response to natural and anthropogenic drivers^53^. Changes in hydraulic, sediment, vegetation, and channel bank characteristics all can lead to channel migration^45,51^. Anthropogenic impacts, such as construction of dams and operation of reservoirs, can also influence channel evolution and have played increasingly important roles in the past several decades^45,52^. Also note that seismic activity may contribute to landslide-induced flooding^59^, changes to river bed morphology^60^, and also rare documented changes to river courses^61^. These effects may become important in localized studies of seismically active zones, however at continental scales the integral effect is expected to be negligible.

Studies focused on understanding river channel changes have documented the lateral migration rates of different rivers, demonstrating large variability in both space and time. The rates of lateral migration most frequently measured are around 1 m per year or less^62^, while the largest lateral migrations measured worldwide were found in the lower reach of the Mississippi River, which was reported as 20 m per year (ref 54) or ranging from <1.0 m per year to >123.0 m per year (ref 48).

Migration rates may be determined from historical surveys or aerial photography, and there has not yet been a systematic compilation or model for the migration rates for the rivers of the entire US. Here, we collected lateral migration rates reported in the literature (Supplementary Table 4). For the two studies with the longest record, Powder River reached the maximum rate at 5 m per year during the 184 year of study^44^, and Klip River in South Africa migrated 160 m during the past 1000 years^55^. The Lower Mississippi River has the largest reported lateral migration of 50 m per year (ref 48).

Also, river migration rates have been found to be related with channel geometry variables, with channel width an important determinant^51^. We listed four empirical relationships developed between lateral migration rate and channel width in Supplementary Table 5. Based on these functions, the estimated migration rate for a river of width 1 km was between 2.93 m per year and 14.8 m per year. Note that the fraction of the total river length with width greater than 1 km is far less than 1% (ref 43), indicating that for the vast majority of river length, migration rates would be much lower than these values.

Therefore, despite the spatial and temporal variation in different rivers, the total migration widths were much smaller than 1 km, the resolution of our analysis, at least within the timescales of our analysis (about two centuries).

The relatively small migration rate values listed above included combined natural and anthropogenic effects. Several studies have analyzed the effect of human activities on migration rates, concluding that 1) migration rates were reduced by about 75% following dam construction^47^; 2) reservoirs can reduce lateral migration rates by factors of 3 to 6 (ref 51); and 3) human engineering, including levees and dam construction, reduced the variability of river width and thus reduced post-dam channel migration^45^.

Overall, based on published migration rates and plausible migration rate ranges derived from migration rate-river width functions, the river course migration rate is much smaller than the resolution of our analysis (1 km). Thus, at this spatial scale the assumption of stationarity of river networks is reasonable. However, the construction of thousands of reservoirs during the last century contributed to changes in the areal coverage of river courses, with corresponding influence on the distance of human populations to rivers. These effects were included in our analysis.

**Supplementary References**

1. Schook, D. M., Rathburn, S. L., Friedman, J. M. & Wolf, J. M. A 184-year record of river meander migration from tree rings, aerial imagery, and cross sections. *Geomorphology* **293**, 227-239 (2017).
2. Richard, G. A., Julien, P. Y. & Baird, D. C. Statistical analysis of lateral migration of the Rio Grande, New Mexico. *Geomorphology* **71**, 139-155 (2005).
3. Ashraf, F. U. & Xiaofeng, L. River meandering prediction: case studies for four rivers in Texas. *World Environmental and Water Resources Congress 2013. Showcasing the Future. Proceedings of the 2013 Congress*, 2009-2019 (2013).
4. Bradley, C. & Smith, D. G. Meandering channel response to altered flow regime - Milk River, Alberta and Montana. *Water Resources Res.* **20**, 1913-1920 (1984).
5. Hudson, P. F. & Kesel, R. H. Channel migration and meander-bend curvature in the lower Mississippi River prior to major human modification. *Geology* **28**, 531-534 (2000).
6. Meitzen, K. M. Lateral channel migration effects on riparian forest structure and composition, Congaree River, South Carolina, USA. *Wetlands* **29**, 465-475 (2009).
7. Black, E. *et al.* Determining lateral migration rates of meandering rivers using fallout radionuclides. *Geomorphology* **123**, 364-369 (2010).
8. Shields, F. D., Simon, A. & Steffen, L. J. Reservoir effects on downstream river channel migration. *Environ. Conserv.* **27**, 54-66 (2000).
9. Wang, S. J., Li, L., Ran, L. S. & Yan, Y. X. Spatial and temporal variations of channel lateral migration rates in the Inner Mongolian reach of the upper Yellow River. *Environmental Earth Sciences* **75**, 14 (2016).
10. Chakraborty, S. & Mukhopadhyay, S. An assessment on the nature of channel migration of River Diana of the sub-Himalayan West Bengal using field and GIS techniques. *Arab. J. Geosci.* **8**, 5649-5661 (2015).
11. Magdaleno, F. & Fernandez-Yuste, J. A. Meander dynamics in a changing river corridor. *Geomorphology* **130**, 197-207 (2011).
12. Rodnight, H., Duller, G. A. T., Tooth, S. & Wintle, A. G. Optical dating of a scroll-bar sequence on the Klip River, South Africa, to derive the lateral migration rate of a meander bend. *Holocene* **15**, 802-811 (2005).
13. Rozo, M. G., Nogueira, A. C. R. & Castro, C. S. Remote sensing-based analysis of the planform changes in the Upper Amazon River over the period 1986-2006. *J. South Am. Earth Sci.* **51**, 28-44 (2014).
14. Brice, J. C. Stream channel stability assessment. Report FHWA/RD-82/021. *US Department of Transportation Federal Highway Administration, Washington, DC* (1982).
15. Macdonald, T. E., Parker, G. & Luethe, D. P. Inventory and Analysis of Stream Meander Problems in Minnesota. *St Anthony Falls Laboratory, University of Minnesota, Minneapolis, MN, USA* (1991).
16. Dai, F. C., Lee, C. F., Deng, J. H., & Tham, L. G. The 1786 earthquake-triggered landslide dam and subsequent dam-break flood on the Dadu River, southwestern China. Geomorphology, 65, 205-221 (2005).
17. Field, M. E., Gardner J. V., Jennings A. E., & Edwards B. D.. Earthquake-induced sediment failures on a 0.25° slope, Klamath River delta, California. Geology 10, 542-546 (1982).
18. Sirovich, L., & F. Pettenati. Source inversion of the 1570 Ferrara earthquake and definitive diversion of the Po River (Italy). *Journal of Geophysical Research: Solid Earth* **120**, 5747-5763 (2015).
19. Lutgens, F. K. & Tarbuck, E. J. *Essentials of Geology*. (Prentice Hall, Englewood Cliffs, 1995).
